# On the External Application of Oil of Turpentine as a Substitute for Quinine in Intermittent Fever, with Reports of Cases

**Published:** 1864-01

**Authors:** 


ART. III.—*On the External Application of Oil of Turpentine as a Substitute for Quinine in Intermittent Fever, with Reports of Cases.* (Communicated.)

At the present time, when the demand for remedies is constantly upon the increase, whilst the supply is actually diminished, and this supply procurable only with difficulty, and at enormous rates, the discovery of what may, to any extent, supply the place of the costly salts of quinia, in the treatment of malarious fevers, becomes of deep interest to the profession.

Very many native remedies have been found valuable adjuncts to this powerful antiperiodic, but as yet no substitute perfectly reliable has been offered. That which at present appears to afford most promise, is the Pinckneya Pubens or Georgia bark, one of the Cinchonæ, found from New River, S. C., along the sea-coast to Florida, in which a considerable amount of cinchonine has been discovered, but of which no complete and perfectly satisfactory analysis has yet been made.

The use of the external application of the oil of turpentine, as a substitute for the internal administration of quinine, not altogether novel, but to which the attention of the Medical Bureau, has been lately called by Surg. Stiles Kennedy, is considered of sufficient importance to invite the attention of the profession at large.

The special mode of action upon which this remedy bases a claim, so far well sustained by experience, is solely and simply by means of a powerful impression upon the nervous centres, especially the central sympathetic system, to interrupt the morbid train of paroxysms established, and set in motion by the malarious poison, and to put the system in a proper condition for the employment of tonics and antiperiodics.

The mode of application, as used and recommended by the officer above mentioned, is as follows: Half an hour before the expected paroxysm, a bandage, wet with the turpentine, is applied around the body at the lower part of the chest, the linen replaced and the outside clothing buttoned. If convenient, the patient should then be placed in blankets: if not, he should be kept in sight, so that he may not remove the bandage.

Surg. K. reports the successful trial of this application without failure in "over thirty cases." The following report, however, from the General Hospital at Guyton, Georgia, selected at random from those which have up to this time been received at the office of the Surgeon-General, presents a fairer average of the results of a series of experiments instituted in several sections of the Confederacy, the blank used being that prescribed and issued from that office for special reports required upon the subject:

*Special report of cases of Intermittent Fever treated by the external application of Turpentine, as a substitute for the internal administration of Quinine. Station—General Hospital, Guyton, Ga. Treated during the month of October,* 1863.

| Name. | Rank. | Regiment. | Type of Fever. | Date and Hour of first Chill. | Date and Hour of Expected Chill. | Time of Application of Turpentine. | How long Application continued. | Total Quantity employed. |
|---|---|---|---|---|---|---|---|---|
| 1. Jesse W. Ward | Private, | 30th Georgia, | Quotidian, | Oct. 21st, 5 P. M. | 22d, 5 P. M. | 4 P. M. | 4 hours, | 1 ounce. |
| 2. John Hanly | Private, | 63d Georgia, | Quotidian, | Oct. 28th, 3 P. M. | 29th, 3 P. M. | 2 P. M. | 3 hours, | 1 ounce. |
| 3. J. B. McCullers | Private, | 32d Georgia, | Quotidian, | Oct. 19th, 7 A. M. | 20th, 7 A. M. | 6 A. M. | 4 hours, | 1 ounce. |
| 4. J. J. Grisham | Private, | Bartow Artil'y, | Tertian, | Oct. 21st, 3 P. M | 23d, 3 P. M | 2 P. M. | 3 hours, | 1 ounce. |
| 5. W. C. Geddens | Private, | 29th Georgia, | Tertian, | Oct. 22d, 10 A. M. | 24th, 10 A. M. | 9 A. M. | 3 hours, | 1 ounce. |
| 6. A. B. Funderbrook | Private, | Engineer corps, | Quotidian | Oct. 23d, 4 P. M. | 24th, 4 P. M. | 3 P. M. | 4 hours, | 1 ounce. |
| 7. S. Wilson | Private, | Engineer corps, | Quotidian, | Oct. 22d, 9 P. M. | 23d, 9 P. M. | 8½ P. M. | 1½ hours, | 1 ounce. |

*Result.*

Case I. Expected chill did not occur, but chills returned November 8th. Tonic bitters used in conjunction. No strangury nor any external injury to the tissues occurred. Subject to chills. Application made around the body below ensiform cartilage and patient kept in bed.

Case 2. Expected chill did not occur nor again return. No other remedies used. No strangury nor external injury to tissues. Was attacked with quotidian October 19th. Turpentine applied in same manner to same part as in case above.

Case 3. Expected chill did not occur nor again return. Liquor potass. arsenit. given for four days after first chill. No strangury nor external injury to the tissues. Subject to chills. Application as above. Neuralgia of left temple afterwards occurred and was relieved by application of turpentine to the part.

Case 4. Expected chill did not occur, but chill returned on the 28th, and treated with quinine, no turpentine being on hand. No bad effects from use of turpentine. Fowler's solution used in conjunction, till diarrhœa appeared. Mode of application as before. Patient subject to chills and disorder of hepatic function.

Case 5. No return of chills. Treatment in conjunction, blisters and the ferri chloride. No strangury nor injury to tissues. Not subject to chills; had remittent in camp. Place and manner of application same as in other cases.

Case 6. Expected chill did not occur, but chill returned on the 30th, and, on re-applying turpentine, did not again return. Fowler's solution after second attack and kept up till bowels became disordered. Subject to chills. Applcation as above.

Case 7. Expected chill did not occur nor again return. Bitter tonics in conjunction. No ill effects from use of turpentine. First attack. Application made around body below ensiform cartilage.

It will be seen from this report, that where there is a probability of the return of the paroxysm on the seventh, or fourteenth day, the repetition of the application is essential to its certain avoidance.

To insure a fair trial of the remedy, the patient should, when it may be found necessary, have the advantage of preliminary treatment, due attention being given to any visceral derangement or obstruction, and to any unnatural condition of the skin.

After the abortion of the paroxysm, tonics and antiperiodics are of course indicated, and among those most available at present, may be mentioned the cold infusion and extract of the Eupatorium and Cornus Floridæ, the powder of the Linodendron, or the compound tincture of the barks of the Linodendron, Salix and Cornus Floridæ. In chronic cases the after employment of Fowler's solution, and of the tincture ferri muriatis, will often, no doubt, prove serviceable.

In conjunction with the special mode of the treatment of malarious fever, herein narrated, the use before the paroxysm, of the warm bitter infusions as diaphoretics, is recommended. Surgeon Hinkley has lately reported several cases from the wayside hospitals at Demopolis, in which the paroxysm was completely prevented by the administration at this period, of the warm infusion of the fresh root of the Verbascum Thapsus (mullein) strength; four ounces of the green root to one pint of water, reduced to one-half by boiling; two ounces of this infusion being given every hour, commencing four hours previous to the expected chill.

It is hoped that this economical mode of treatment, the results of which have been thus far so very satisfactory, will still be persevered in, and that substantial reports of cases will be promptly forwarded, in order that a still more satisfactory conclusion may be reached, and interesting statistical information on the subject be to a larger extent given to the profession.

